# Expression and Functional Characterization of a Novel Antimicrobial Peptide: Human Beta-Defensin 118

**DOI:** 10.1155/2020/1395304

**Published:** 2020-11-09

**Authors:** Qian Lin, Kunhong Xie, Daiwen Chen, Bing Yu, Xiangbing Mao, Jie Yu, Junqiu Luo, Ping Zheng, Yuheng Luo, Hui Yan, Jun He

**Affiliations:** ^1^Institute of Animal Nutrition, Sichuan Agricultural University, Chengdu, Sichuan 611130, China; ^2^Key Laboratory of Animal Disease-Resistant Nutrition, Sichuan Province, Chengdu 611130, China

## Abstract

**Purpose:**

*β*-Defensin 118 (DEFB118) is a novel host defense peptide (HDP) identified in humans. To evaluate its potentials for future utilization, the DEFB118 gene was expressed in *Escherichia coli* (*E. coli*) and the recombinant protein was fully characterized.

**Methods:**

The DEFB118 protein was obtained by heterologous expression using *E. coli* Rosetta (DE3). Antibacterial activity of DEFB118 was determined by using various bacterial strains. IPEC-J cells challenged by *E. coli* K88 were used to determine its influences on inflammatory responses.

**Results:**

The *E. coli* transformants yielded more than 250 *μ*g/mL DEFB118 protein after 4 h induction by 1.0 mM IPTG. The DEFB118 was estimated by SDS-PAGE to be 30 kDa, and MALDI-TOF analysis verified that it is a human *β*-defensin 118. Importantly, the DEFB118 showed antimicrobial activities against both Gram-negative bacteria (*E. coli K88* and *E. coli DH5α*) and Gram-positive bacteria (*S. aureus* and *B. subtilis*), with a minimum inhibitory concentration (MIC) of 4 *μ*g/mL. Hemolytic assays showed that DEFB118 had no detrimental impact on cell viability. Additionally, DEFB118 was found to elevate the viability of IPEC-J2 cells upon *E. coli* K88 challenge. Moreover, DEFB118 significantly decreased cell apoptosis in the late apoptosis phase and downregulated the expression of inflammatory cytokines such as IL-1*β* and TNF-*α* in IPEC-J2 cell exposure to *E. coli* K88.

**Conclusions:**

These results suggested a novel function of the mammalian defensins, and the antibacterial and anti-inflammatory properties of DEFB118 may allow it as a potential substitute for conventionally used antibiotics or drugs.

## 1. Introduction

In the last decades, antibiotics have been widely used as a therapeutic medicine to control various infectious diseases [1–3]. However, their continuous use not only leads to serious environmental pollution but also increases the risk of developing drug resistance [4–8]. Therefore, developing novel substitutes for conventionally used antibiotics has attracted considerable research interest worldwide [9–12]. Host defense peptides (HDPs), a major subclass of the antimicrobial peptide (AMP) family, are expressed in a variety of epithelial tissues and cells [13, 14]. Previous studies indicated that HDPs can disrupt the bacterial membranes by forming nonspecific electrostatic interactions with membrane lipids and therefore can protect the host from a broad range of pathogens including bacteria, virus, and fungus [15–17]. Considering the fact that bacteria are less able to develop resistance to HDPs than to traditional antibiotics [17], the administration of HDPs is a potentially novel therapeutic strategy for infectious diseases and may present a promising alternative to the traditional antibiotics.

Defensins comprise an important family of HDPs for mammalian animals. According to distribution patterns of the intramolecular disulfide bonds, defensins are classified into *α*- and *β*-forms [18, 19]. The *α*-defensins are expressed in neutrophils and paneth cells, whereas the *β*-defensins are usually expressed in the epithelia. Recent studies indicated that the *β*-defensins have multidirectional biological properties, including antiviral, antibacterial, and anti-inflammatory effects [20, 21]. For instance, *β*-defensin 114 was found to show antimicrobial activities against *E. coli* DH5*α* and *E. coli* K88 [22], while *β*-defensin 129 was reported to attenuate intestinal inflammation and epithelial atrophy in rat exposure to bacterial endotoxin [14]. The human *β*-defensin 118 (DEFB118) is a novel identified HDP, which is present in the epithelial cells of different ducts and most abundant in the caput epithelium, where it is present in the lumen and located on sperm [23]. A previous study indicated that the DEFB118 caused rapid permeabilization of both outer and inner membranes of *E. coli* and striking morphological alterations in the bacterial surfaces [24]. Therefore, the DEFB118 may contribute to epididymal innate immunity and protect the sperm against attack by microorganisms in the male and female reproductive tracts.

Although numerous studies indicated a critical role of *β*-defensins in the host defense against exogenous pathogens, the functions of DEFB118 protein have not been fully characterized. Moreover, the production of DEFB118 is still not commercially feasible because of its high cost. In the present study, we describe the cloning and expression of the DEFB118 gene by a pET expression vector in *E. coli*. In addition, the antimicrobial and anti-inflammatory activities of the DEFB118 protein were fully characterized.

## 2. Materials and Methods

### 2.1. Strains and Vectors

The *E. coli* DH5*α* and *E. coli* Rosetta (DE3) strains were purchased from TIANGEN (China). *E. coli* K88 was kindly provided by Professor Lianqiang Che, Institute of Animal Nutrition, Sichuan Agricultural University. *Salmonella typhimurium* ATCC14028 (*S. typhimurium*), *Staphylococcus aureus* CICC23656 (*S. aureus*), and *Bacillus subtilis* (*B. subtilis*) were kindly provided by Professor Qigui Yan, College of Animal Science and Technology, Sichuan Agricultural University. The pET32a (+) was purchased from Invitrogen.

### 2.2. Plasmid Construction

DEFB118 gene was obtained by complete gene synthesis (Beijing Cycle-Tech Biotechnology Co., Ltd., China). The company provides a cloned strain of DH5*α*-PMD19-DEFB118 containing the target gene DEFB118. And sequencing was identified in Shenggong Biological Engineering Co., Ltd. Sequencing results were compared by software DNAMAN. The plasmid PMD19-DEFB118 was extracted by using Plasmid Mini Kit I (Omega, America) according to the manufacturer's recommendations. The plasmid pMD19-DEFB118 and expression vector pET32a (+) were double-digested with EcoRI and NotI enzymes (Takara, Japan) at 37°C for 4 h. After purification by agarose gel electrophoresis, the isolated DNA fragments were ligated by T4 DNA ligase (Takara, Japan). And then, the ligated product was transformed into *E. coli* DH5*α* cells using the heat shock method and plated on LB agar containing kanamycin (50 *μ*g/mL). The positive colonies were randomly picked, then confirmed by restriction enzyme digestion and sequenced by Sangon Biotech (Shanghai, China).

### 2.3. Expression of DEFB118 in *E. coli*

For expression, plasmid pET32a (+)-DEFB118 gene was transformed into *E. coli* Rosetta (DE3) cells using a heat shock method. And then, positive bacterial colonies were confirmed as the methods of construction of the expression vector. The selected positive bacteria were incubated, once OD 600 reached 1.0; then, 1.0 mM isopropyl *β*-d-1-thiogalactoside (IPTG) was added to induce protein expression. After incubation for 4 h at 28°C, bacterial cells were harvested by centrifugation at 8000 × g for 10 min at 4°C and lysis by lysis buffer (500 mM NaCl, 20 mM Tris, 0.1% Triton X-100, 1 mM PMSF, Lysozyme 0.2 mg/mL, 10 U/mL DNase (pH 7.5)) for 30 min at 4°C. Then, schizolytic cells were sonicated (4 s pulse and 8 s interval; 30 cycles; Sonics Vibra-Cell, USA) and centrifuged at 15,000 × g for 30 min at 4°C. The supernatant was collected and stored at -80°C until analysis.

### 2.4. Affinity Purification

The supernatant obtained above was filtered by 0.22 *μ*m filter and then applied to Ni 2+-IDA column (Sangon Biotech, China) and purified according to specification. Briefly, 10 resin volumes of Binding Buffer (50 mM NaH_2_PO_4_, 300 mM NaCl, pH 8.0) were added to wash away the impure protein, and then, 5 resin volumes of Elution Buffer (50 mM NaH_2_PO_4_, 300 mM NaCl, 150 mM imidazole, pH 8.0) were added to elute the DEFB118 from the column. Then, protein concentration was quantified with the BCA assay (Beyotime, China). The purified DEFB118 was ran on 12% SDS-PAGE. The rest was stored at −80°C to analyze antimicrobial activities.

### 2.5. Antimicrobial Activity Assays

The minimal inhibitory concentration (MIC) of purified DEFB118 was measured by the microtiter broth dilution method [25]. *E. coli* DH5*α*, pathogenic *E. coli* K88+, *S. typhimurium*, *S. aureus*, and *B. subtilis* were grown to 0.4 OD 600 nm at 37°C in LB; *Streptococcus* was grown to 0.4 OD 600 nm at 37°C in THY (Todd-Hewitt+yeast extract). The target cell culture was diluted to 1 × 10^5^ CFUs/mL with the same media, respectively. A total of 100 *μ*L of DEFB118 and 100 *μ*L of cell suspension were added into each well. The activity of DEFB118 was tested over a concentration range of 512, 256, 128, 64, 32, 16, 8, 4, 2, and 1 mg/L, and all assays were tested in triplicate. Bacterial plates were incubated at 37°C for 16 h, and the absorption of cell culture was recorded at 600 nm. MIC was defined as the lowest concentration of peptide at which there was no change in optical density.

### 2.6. Hemolytic Activity Assay

Hemolytic activity of DEFB118 was determined as described earlier [26]. In brief, erythrocytes from heparinized pig blood were washed thrice with cold PBS (pH 7.2) and resuspended to a concentration of 4% in saline. Erythrocytes were treated with different concentrations of DEFB118 (200 *μ*L) in a 96-well plate and incubated at 37°C for 1 h. The plate was centrifuged at 1000 rpm for 5 min, and supernatants were transferred to a fresh plate. Absorbance at 414 nm of saline and 0.1% Triton X-100-treated erythrocytes served as 0 and 100% hemolysis controls, respectively.

### 2.7. Cell Culture

Intestinal porcine epithelial cells (IPEC-J2) were cultured in a 75 cm^2^ cell culture flask in DMEM-F12 with 10% FBS, 100 U/mL penicillin, and 100 *μ*g/mL streptomycin. 1 × 10^5^ cells/well were seeded in 12-well plates and grown to ~60% confluence at 37°C in a CO_2_ incubator (5% *v*/*v*), then incubated with antimicrobial peptides DEFB118 for 12 h (DEFB118, DEFB118+*E. coli* K88). Cells were challenged with 1 × 10^6^ CFU/well *E. coli* K88 for  h (DEFB118+*E. coli* K88, *E. coli* K88); control cells were cultured in a culture medium of 2% serum (without any antibiotics) without any treatment. Total cellular RNA was collected using RNAiso Plus (Takara, Dalian, China).

### 2.8. Cytotoxicity Assay

The cytotoxicity of DEFB118 was measured according to a previous study [27]. Briefly, IPEC-J2 cells were cultured in DMEM-F12 with 10% FBS, 100 U/mL penicillin, and 100 *μ*g/mL streptomycin for 48 h and then resuspended to 10^5^ cells/mL in FBS-free DMEM-F12 media. A volume of 100 *μ*L of cells was aliquoted into sterile flat-bottomed 96-well plates (Corning, USA). The DEFB118 was added to the cells and incubated at 37°C/5% CO_2_ for 24 h (DEFB118, DEFB118+*E. coli* K88). Then, cells were challenged with 1 × 10^6^ CFU/well *E. coli* K88 for 1 h (DEFB118+*E. coli* K88, *E. coli* K88); control cells were cultured in complete medium without any treatment. Cell viability was evaluated with the CCK8 assay (Beyotime, Shanghai, China) according to the manufacturer's instructions.

### 2.9. Adhesion Experiment

1 × 10^5^ cells/well were seeded in 6-well plates and grown to ~60% confluence at 37°C in a CO_2_ incubator (5% *v*/*v*), then incubated with antimicrobial peptides DEFB118 for 12 h (DEFB118+*E. coli* K88). Cells were challenged with 1 × 10^6^ CFU/well *E. coli* K88 for 1 h (DEFB118+*E. coli* K88, *E. coli* K88). The supernatant was aspirated, and the floating cells and *E. coli* K88 were washed with 0.01 M PBS. The cells were lysed with sterile distilled water, diluted in a series of lysates, and coated in MacConkey agar medium. The number of colonies grown indicated the number of adherents of *E. coli* K88 to epithelial cells.

### 2.10. Assessment of Apoptosis by Flow Cytometry

In order to evaluate the protective effect of DEFB118 against *E. coli* K88, IPEC-J2 cells were grown to ~60% confluence at 37°C in a CO_2_ incubator (5% *v*/*v*), then incubated with DEFB118 for 12 h (DEFB118, DEFB118+*E. coli* K88). Cells were challenged with 1 × 10^6^ CFU/well *E. coli* K88 for 2.5 h (DEFB118+*E. coli* K88, *E. coli* K88); control cells were cultured in a culture medium of 2% serum (without any antibiotics) without any treatment. Treated cells were harvested and labeled with an anti-Annexin V-FITC Apoptosis Detection Kit (BD Biosciences, USA). Floating cells were collected; then, attached cells were washed with 0.01 M PBS and trypsinized for 2 min. Finally, trypsinized cells and floating cells were added together to centrifugate at 350 g for 10 min, then stained with Annexin V-FITC and propidium iodide (PI). The intensity of the markers was examined by flow cytometry (FACSCanto II, BD Biosciences, USA). All flow cytometric data were analyzed using FlowJo software (BD Biosciences, USA).

### 2.11. RNA Extraction and RT-PCR

IPEC-J2 cells were harvested, and the total RNA was extracted using RNAiso Plus (Takara, Dalian, China) according to the manufacturer's instructions. The quantity and quality of the isolated RNA were determined by absorbance at 260 and 280 nm [28]. And then, cDNA was synthesized using a Reverse Transcriptase kit (Takara, Dalian, China). Briefly, quantitative PCR was performed by the QuantStudio 6 Flex Real-Time PCR detection system (Applied Biosystems, Foster City, CA, USA) with a total of 10 *μ*L of assay solution containing 5 *μ*L SYBR Green mix (Takara), 0.2 *μ*L Rox, 3 *μ*L deionized H_2_O, 1 *μ*L cDNA template, and 0.4 *μ*L each of forward and reverse primers (Qingke, China). The relative gene expressions compared with the housekeeping gene *β*-actin were calculated by 2^-CT^ [29].

### 2.12. Statistical Analysis

All statistical analysis was performed using SPSS 21.0 software. Data were expressed as the mean ± standarderrorofthemean(SEM). Statistical analysis of the treatment of IPEC-J2 and cytotoxicity was carried out using two-way ANOVA followed by Duncan's multiple comparisons test. Image was produced using GraphPad Prism software (Version 7, GraphPad Software Inc., CA, USA).

## 3. Results

### 3.1. Comparison of the DEFB118 Nucleotide Sequences

**Figure 1:**
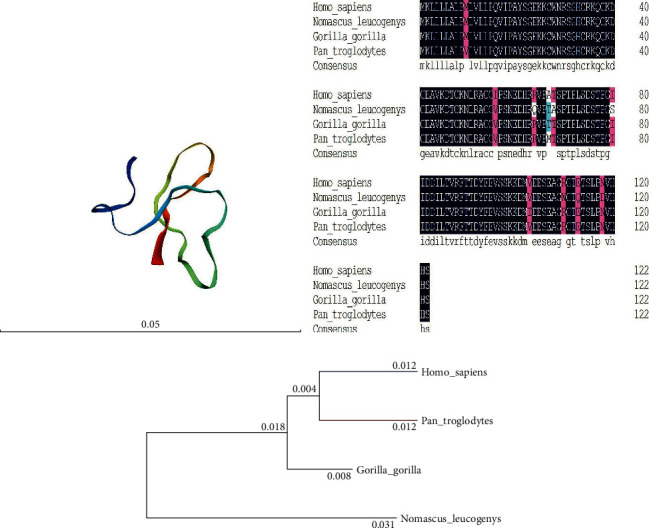
Phylogenetic analysis of beta-defensin 118. (a) Prediction model of DEFB118; (b) phylogenetic analysis of beta-defensin 118 in Homo sapiens, Pan troglodytes, Gorilla gorilla, and Nomascus leucogenys was performed by DNAMAN 8.0; (c) amino acid sequences of beta-defensin 118 in *Homo sapiens*, *Pan troglodytes*, *Gorilla gorilla*, and *Nomascus leucogenys* were aligned by DNAMAN 8.0.

Blast analysis of the synthesized DEFB118 sequence was performed by using the DNAMAN 8.0. Results showed that the synthesized DEFB118 sequence was consistent with the published sequence (Fig. S1). Both contain a 372 bp open reading frame, which encodes a 123-amino acid DEFB118 mature protein. Structural analysis by using the “SWISS model” showed that the DEFB118 protein exhibited a classic beta-ring conformation (Figure 1(a)). Amino acid sequence analysis showed that the amino acid sequence of DEFB118 is highly conserved (Figure 1(b)). The human DEFB118 sequence is more than 97% identical to the sequences obtained from *Pan troglodytes*, *Gorilla gorilla*, and *Nomascus leucogenys*. Phylogenetic tree analysis showed that the human DEFB118 is close to that of *Gorilla gorilla* (Figure 1(c)).

### 3.2. Expression and Purification of the Recombinant DEFB118

**Figure 2:**
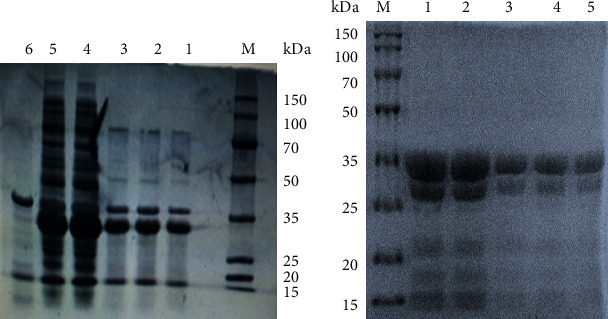
SDS-PAGE analysis of DEFB118 produced by *E. coli* Rosetta. (a) SDS-PAGE of DEFB118 from E. coli Rosetta. M protein markers (DL 150 kDa); Lane 1: *E. coli* Origami B (DE3)-pET32a (+) induced by 1 mmol/L IPTG for 10 h at 28°C; Lane 2: *E. coli* Origami B (DE3)-pET32a (+) induced by 1 mmol/L IPTG for 8 h at 28°C; Lane 3: *E. coli* Origami B (DE3)-pET32a (+) induced by 1 mmol/L IPTG for 6 h at 28°C; Lane 4: *E. coli* Origami B (DE3)-pET32a (+) induced by 1 mmol/L IPTG for 4 h at 28°C; Lane 5: *E. coli* Origami B (DE3)-pET32a (+) induced by 1 mmol/L IPTG for 2 h at 28°C; Lane 6: *E. coli* Origami B (DE3)-pET32a (+) (noninduced). (b) Purification of DEFB118. M protein markers (DL 150 kDa); Lanes 1-2 DEFB118 (1.2 mg/mL) purified by Ni 2+-IDA affinity chromatography; Lanes 3-5 DEFB118 (0.6 mg/mL) purified by Ni 2+-IDA affinity chromatography.

**Figure 3:**
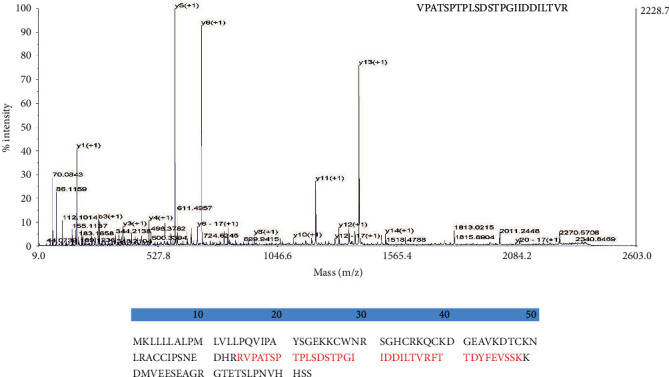
Mass spectrometry identification of DEFB118. The purified target band in Figure 2(b) was collected, and the amino acid sequence of DEFB118 was identified by MALDI-TOF/TOF. (a) Peak figure of amino acid fragments; (b) through searching UniProt-*Homo-sapiens*, DEFB118 sequence had a match with NP_473453.1 (shown in red).

The DEFB118 gene with two designated restriction enzyme sites (EcoRI/NotI) was artificially synthesized. The two restriction enzyme sites allow directional cloning of the DEFB118 gene into the pET32a expression vector. A 312 bp fragment was observed after double digestion of recombinant plasmid with the two restriction enzymes (Fig. S2a). The recombinant plasmids were transformed into the *E. coli* Rosetta (DE3), and the positive clones were selected by PCR (Fig. S2B). The most desired strain was chosen for small-scale induction by using 1 mmol/L IPTG at 28°C. We found that the induction time significantly affected the expression level. As shown in Figure 2(a), *E. coli* achieved a maxima yield of the DEFB118 protein after 4 h induction (more than 250 *μ*g/mL). The molecular weight of recombinant DEFB118 was estimated by SDS-PAGE to be 30 kDa (Figures 2(a) and 2(b)). The crude protein collected from ultrasonically disrupted bacteria was purified by using Ni^2+^-IDA affinity chromatography (Figure 2(b)). The target band was collected, and amino acid sequence of DEFB118 was identified by using mass spectrometry (MALDI-TOF/TOF). As shown in Figure 3, the protein sequence of the recombinant DEFB118 protein 100% matches the sequence of human DEFB118 (NP_473453.1).

### 3.3. Antibacterial Activity of the DEFB118

The antibacterial activities of DEFB118 were investigated by using Gram-negative and Gram-positive bacteria strains. As shown in Table 1, DEFB118 showed strong antibacterial activity against Gram-negative bacteria such as the *E. coli K88* and *E. coli DH5α* with a MIC of 4 mg/L. DEFB118 also showed antibacterial activity against the *S. typhimurium* (with a MIC of 8 mg/L). Moreover, the DEFB118 showed strong antibacterial activities against Gram-positive bacteria such as the *S. aureus* and *B. subtilis* with a MIC of 4 *μ*g/mL.

### 3.4. Hemolytic Activity of the DEFB118

**Figure 4:**
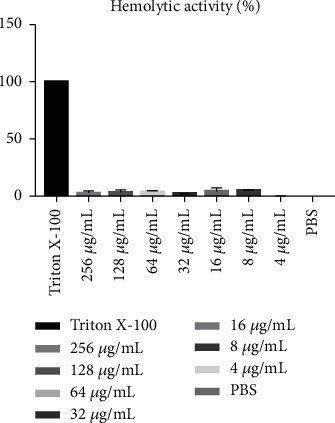
Hemolytic activity of recombinant of DEFB118. DEFB118: 256 *μ*g/mL DEFB118; Triton X-100: 1% Triton X-100; PBS: 10 mM PBS (pH 7.3).

Erythrocytes were collected from fresh porcine blood and incubated with different concentrations (0–256 mg/L) of DEFB118 for 1 h. As compared to the Triton X-100, the DEFB118 showed no significant hemolytic activity at all concentrations (Figure 4).

### 3.5. Influences of DEFB118 on Cell Viability, Apoptosis, and Inflammatory Response in IPEC-J2 Cells

**Figure 5:**
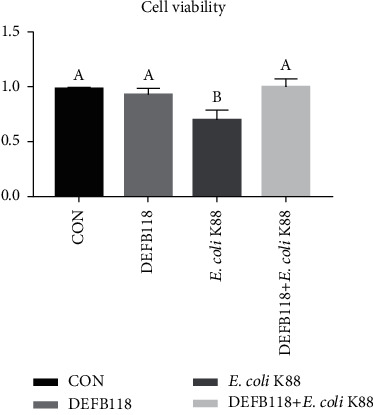
Influence of DEFB118 on *E. coli* K88-induced cell viability in IPEC-J2 cells. IPEC-J2 was determined by incubation with CCK8 for 1 h after different treatments. (a, b) Values within a column differ if they do not share a common superscript (*P* < 0.05).

**Figure 6:**
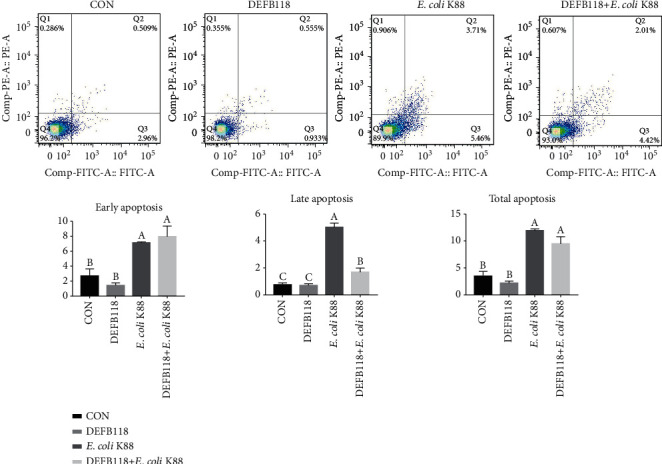
Influence of DEFB118 on *E. coli* K88-induced apoptosis in IPEC-J2 cells. Cell distribution analysis of apoptosis of IPEC-J2 cells treated with DEFB118, *E. coli* K88, and DEFB118 plus *E. coli* K88. In each diagram, Q1 represents the percentage of nonviable, necrotic cells, Q2 represents the percentage of late apoptotic IPEC-J2 cells, Q3 represents the percentages of early apoptotic IPEC-J2 cells, and Q4 represents the percentage of live IPEC-J2 cells. The statistical analysis of cell distribution data among samples; total apoptotic cells included Q2 with Q3. (a–c) Values within a column differ if they do not share a common superscript (*P* < 0.05).

**Figure 7:**
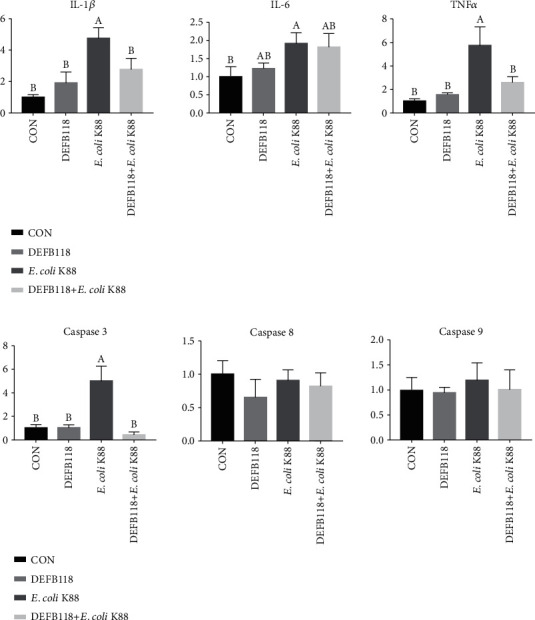
Influence of DEFB118 on *E. coli* K88-induced inflammatory responses in IPEC-J2 cells. Total RNA was extracted from IPEC-J2 cells, and the expression of related genes was measured by real-time fluorescence PCR. The target gene mRNA expression level was calculated using the 2^–*ΔΔ*Ct^ method: (a) proinflammatory cytokine; (b) apoptotic factor. (A, B) Values within a column differ if they do not share a common superscript (*P* < 0.05). IL-1*β*: interleukin 1 beta; IL-6: interleukin 6; TNF*α*: tumor necrosis factor alpha.

As shown in Figure 5, *E. coli* K88 challenge decreased the viability of the IPEC-J2 cells. However, DEFB118 treatment significantly elevated the cell viability (*P* < 0.05). Moreover, DEFB118 can inhibit the adhesion of *E. coli* K88 on the IPEC-J2 cells (*P* < 0.05) (Fig. S3). As compared to the control group, *E. coli* K88 challenge significantly elevated the apoptosis rate in the IPEC-J2 cells (Figure 6). However, DEFB118 treatment significantly decreased the late apoptosis rate in the *E. coli* K88-challenged cells (*P* < 0.05). Interestingly, *E. coli* K88 challenge significantly elevated the expression levels of inflammatory cytokines such as IL-1*β*, IL-6, and TNF-*α* in the IPEC-J2 cells (Figure 7(a)). However, DEFB118 treatment downregulated their expression levels in the *E. coli* K88-challenged cells (*P* < 0.05). Moreover, DEFB118 treatment significantly downregulated the expression level of caspase 3 in the *E. coli* K88-challenged cells (*P* < 0.05).

## 4. Discussion

In the last decades, the use and misuse of antibiotics have led to the development of antibiotic resistance (AMR), which is one of the biggest threats to global public health [30]. The World Health Organization (WHO) predicts that there will be 10 million deaths due to AMR in 2050 [31]. Therefore, substitutes for conventionally used antibiotics have attracted considerable research interest worldwide. Defensins are a family of host defense peptides present in vertebrates, invertebrates, and plants. Currently, the *β*-defensins have attracted considerable research interest since it has been reported to show a broad-spectrum antimicrobial activity and participate in the regulation of immune functions [32–34]. DEFB118 is a newly identified human beta-defensin, which is highly expressed in the epithelial cells of different ducts and most abundant in the caput epithelium [23]. However, the exact role of DEFB118 is poorly understood. Moreover, direct isolation of the DEFB118 from human tissues is not commercially feasible because of its low quantity [35]. In the present study, the DEFB118 was obtained by using heterologous expression, and the recombinant DEFB118 was purified and fully characterized.

The recombinant DEFB118 was estimated by SDS-PAGE to be 30 kDa, and MALDI-TOF analysis indicated that its amino acid sequence is consistent with human beta-defensin 118. It is a well-known fact that the culture conditions such as the induction times and temperatures will affect the yield of protein expression for heterologous expression systems [36]. In this study, the highest yield of DEFB118 in *E. coli* was observed after 4 h IPTG induction. This is different from a previous study which achieved a maximal expression after 2 h induction [36]. The difference may result from the use of different bacteria strains [36]. Interestingly, antimicrobial activity assays showed that DEFB118 has significant antimicrobial activity against both the Gram-positive bacteria (*S. aureus* and *B. subtilis*) and Gram-negative bacteria (*E. coli K88* and *E. coli DH5α*). The MIC for DEFB118 against *S. aureus* and *E. coli K88* was 4 mg/L, which is lower than beta defenses obtained in previous study [22]. These results are also consistent with a previous report on the DEFB118, and both results indicated that the DEFB118 had a broad spectrum of antibacterial activities [36]. There are many articles about the mechanism of human *β*-defensins killing bacteria. Most defensins kill bacteria by destroying the bacterial biofilm structure of bacteria [37, 38]. In the previous study of DEFB118, we found that DEFB118 destroyed the normal morphology and structure of bacteria by increasing the permeability of the bacterial membrane, causing bacterial death [36]. Importantly, hemolytic assays showed that DEFB118 had no detrimental impact on cell viability, indicating that it is safe for human use and may be tentatively used as a substitute for conventionally used antibiotics.

In addition to their antibacterial activities, evidence is accumulating to show that the *β*-defensins can also function as an immunomodulator for mammalian animals. For instance, human *β*-defensin 1, *β*-defensin 2, and *β*-defensin 3 were found to have both the anti-inflammatory and immunoregulatory functions [39–42]. Enterotoxigenic *Escherichia coli* (ETEC) adheres to the intestinal epithelium and induces severe diarrhea and intestinal inflammation [43]. In this study, we found that DEFB118 can inhibit the adhesion of *E. coli* K88 to intestinal epithelial cells. This may be the reason why DEFB118 can protect the body against *E. coli* K88 infection. Moreover, we explored the influence of DEFB118 on inflammatory responses in the intestinal epithelial cell exposure to ETEC K88 [44]. We found that *E. coli* K88 challenge significantly decreased the cell viability and elevated the apoptosis rate in the PIEC-J2 cells. This is consistent with previous studies that microbial infections or stresses increase the apoptosis of the intestinal epithelial cells [45]. Interestingly, DEFB118 significantly decreased the apoptosis in the ETEC K88-challenged cells. This is probably due to the downregulation of several critical inflammatory cytokines such as the IL-1*β* and TNF-*α*. Previous study indicated that overproduction of inflammatory cytokines has resulted in changes in whole-body metabolism and disruption of the tissues such as the muscle and intestinal mucosa [46, 47]. Moreover, both the IL-1*β* and TNF-*α* can induce cell apoptosis via the intrinsic mitochondrial apoptotic pathway [48, 49]. In the present study, ETEC K88 challenge significantly elevated their expression levels in the PIEC-J2 cells. However, DFEB118 treatment resulted in significant downregulation of the two critical inflammatory cytokines. The result is also consistent with previous studies on different animal species [14, 50].

Caspases are proteolytic enzymes that mediate programmed cell death (apoptosis) and are highly conserved among different species [51]. The family of caspases can be further divided into the initiator (caspases 8, 9, and 10) and executioner (caspases 3, 6, and 7) [52]. Among these executioners, caspase 3 is extremely important as both intrinsic and extrinsic pathways converge at caspase 3 [53]. In this study, *E. coli* K88 challenge significantly elevated the expression levels of caspase 3 in the PIEC-J2 cells, which was consistent with a previous study on piglets [54]. However, DEFB118 can downregulate the expression levels of caspase 3. This is probably due to the downregulation of inflammatory cytokines (i.e., TNF-*α*) since they were reported to induce apoptosis via activation of the caspase system [48, 49].

## 5. Conclusions

The DEFB118 shows a broad spectrum of antimicrobial activities and few hemolytic activity and cytotoxicity. Additionally, DEFB118 increases the cell viability in the intestinal epithelial cell exposure to *E. coli* K88, which was associated with decreased cell apoptosis and downregulation of inflammatory cytokines. The antibacterial and anti-inflammatory properties of DEFB118 may allow it as a potential substitute for conventionally used antibiotics or drugs.

## Figures and Tables

**Table 1:** MIC of DEFB118 produced by E. coli Rosetta (DE3).

Strain	MIC (*μ*g/mL)
*Gram-negative bacteria*	
*E. coli DH5*	4
*Pathogenic E. coli K88*	4
*Salmonella typhimurium CICC14028*	8
*Gram-positive bacteria*	
*Streptococcus*	32
*Staphylococcus aureus CICC23656*	4
*Bacillus subtilis*	4

MIC: minimal inhibitory concentration.

## Data Availability

The datasets used and/or analyzed during the current study are available from the corresponding author on reasonable request.

## Supplementary Materials

Supplementary 1 Fig. S1: strategy of cloning of DEFB118 gene. The CDS sequence without signal peptide of DEFB118 was cloned; *EcoRΙ* and *NotΙ* were inserted in 5′ and 3′, respectively.

Supplementary 2 Fig. S2: results from agarose gel electrophoresis (EcoRI/Not I digestion map). (a) Digestion of the recombinant expression vectors. Lane M1 DNA marker (DL 10000), Lane 1 DEFB118 (without digestion). Lane 2 DEFB118 (digestion with EcoRI and NotI). Lane M2 DNA marker (DL 2000). (b) RT-PCR of DEFB118. Lane M DNA marker (DL 2000), Lanes 1-4 DEFB118 (production of five tubes of reaction solution).

Supplementary 3 Fig. S3: influence of DEFB118 on adhesion of *E. coli* K88 to IPEC-J2 cells. MacConkey agar plate was coated with serial diluted cell lysate. The number of colonies grown indicated the number of adherents of *E. coli* K88 to epithelial cells. (a, b) Values within a column differ if they do not share a common superscript (*P* < 0.05).
