# Supplementary material for: Expression and Functional Characterization of a Novel Antimicrobial Peptide: Human Beta-Defensin 118
Source: Biomed Res Int. 2020 Nov 9;2020:1395304. doi: 10.1155/2020/1395304 (PMC7673234; doi:10.1155/2020/1395304)
Supplement: Supplementary 1 — Fig. S1: strategy of cloning of DEFB118 gene. The CDS sequence without signal peptide of DEFB118 was cloned; EcoRΙ and NotΙ were inserted in 5′ and 3′, respectively. [file 1395304.f1.pdf]

EcoR I

GAATTCTATAGTGGTGAAAAAAAATGCTGGAACAGAT
CAGGGCACTGCAGGAAACAATGCAAAGATGGAGAA
GCAGTGAAAGATACATGCAAAAATCTTCGAGCTTGCT
GCATTCCATCCAATGAAGACCACAGGCGAGTTCCTG
CGACATCTCCCACACCCTTGAGTGACTCAACACCAG
GAATTATTGATGATATTTTAACAGTAAGGTTCACGACA
GACTACTTTGAAGTAAGCAGCAAGAAAGATATGGTT
GAAGAGTCTGAGGCGGGAAGGGGAACTGAGACCTC
TCTTCCAAATGTTCACCATAGCTCATGA***GCGGCCGC***

Not I
