# Supplementary figures and images for: Expression and Functional Characterization of a Novel Antimicrobial Peptide: Human Beta-Defensin 118
Source: Biomed Res Int. 2020 Nov 9;2020:1395304. doi: 10.1155/2020/1395304 (PMC7673234; doi:10.1155/2020/1395304)

M1 1 2 M2

bp

10000-  
7000-  
4000-  
2000-  
1000-  
500-  
250-

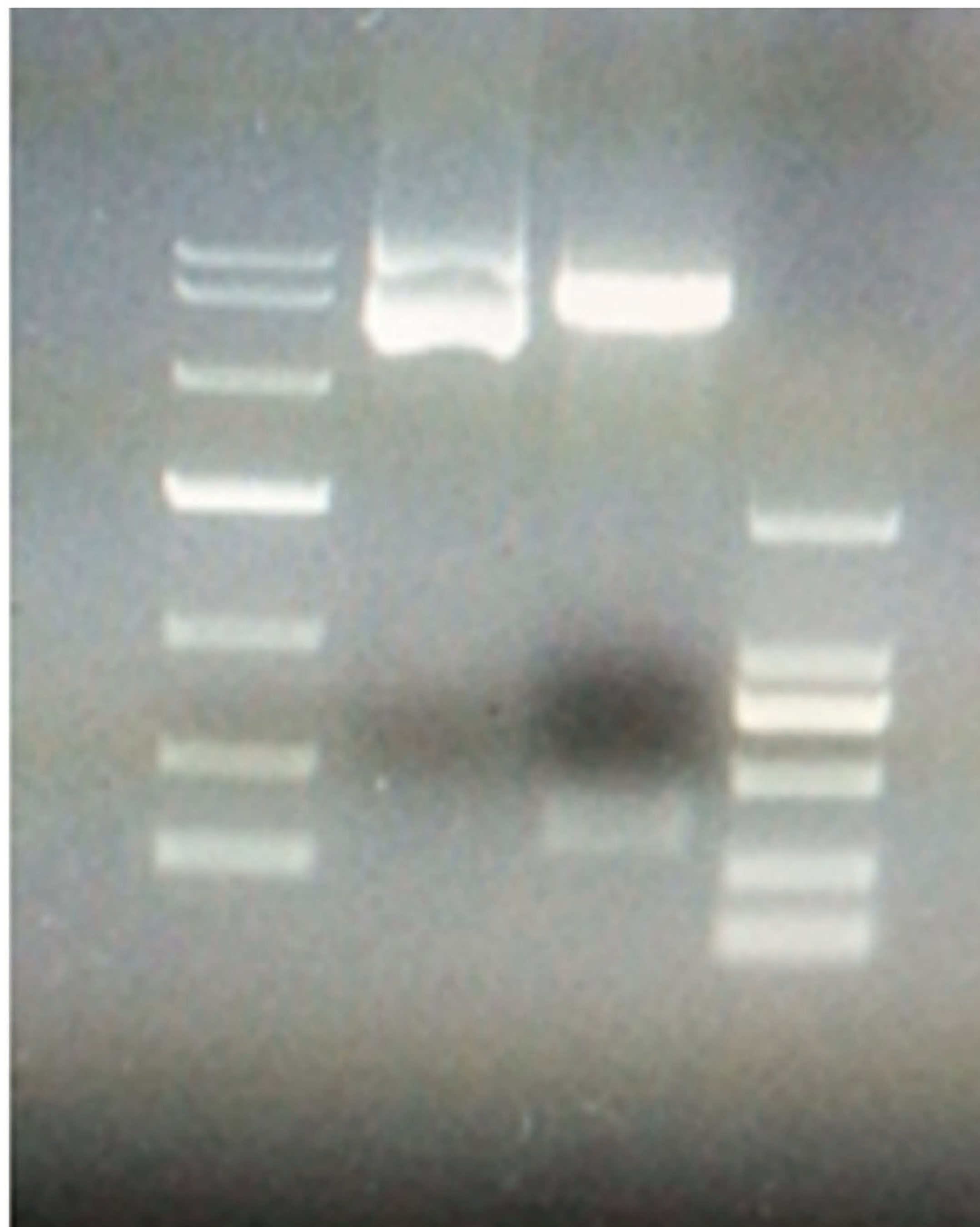

bp

-2000  
-1500  
-750  
-500  
-250  
-100

M 1 2 3 4

bp  
2000-  
1000-  
750-  
500-  
250-  
100-

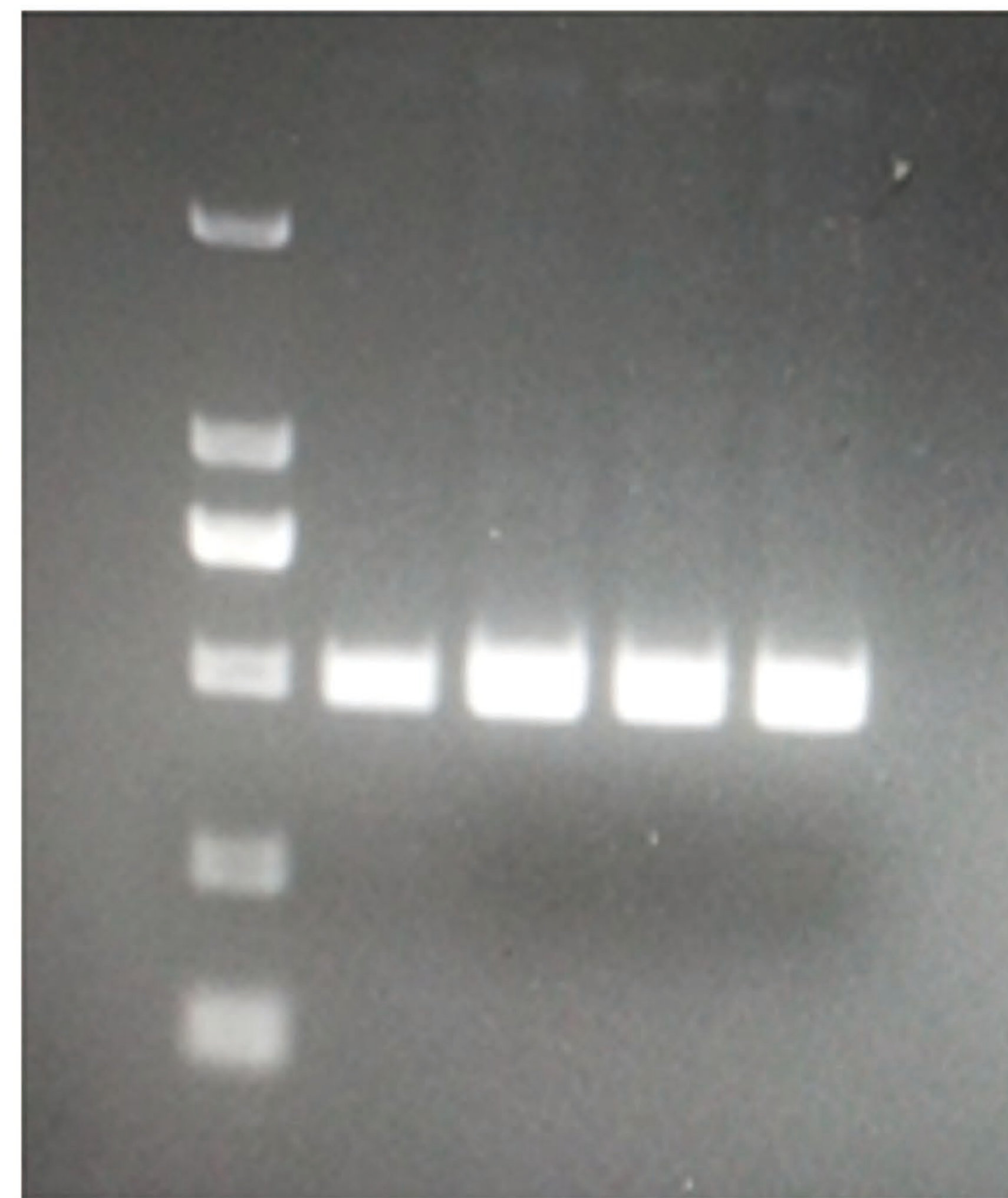

Supplement: Supplementary 2 — Fig. S2: results from agarose gel electrophoresis (EcoRI/Not I digestion map). (a) Digestion of the recombinant expression vectors. Lane M1 DNA marker (DL 10000), Lane 1 DEFB118 (without digestion). Lane 2 DEFB118 (digestion with EcoRI and NotI). Lane M2 DNA marker (DL 2000). (b) RT-PCR of DEFB118. Lane M DNA marker (DL 2000), Lanes 1-4 DEFB118 (production of five tubes of reaction solution). [file 1395304.f2.pdf]

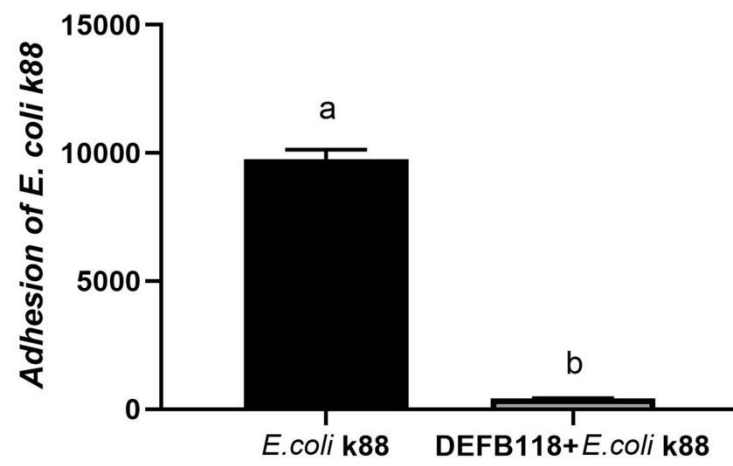

Supplement: Supplementary 3 — Fig. S3: influence of DEFB118 on adhesion of E. coli K88 to IPEC-J2 cells. MacConkey agar plate was coated with serial diluted cell lysate. The number of colonies grown indicated the number of adherents of E. coli K88 to epithelial cells. (a, b) Values within a column differ if they do not share a common superscript (P < 0.05). [file 1395304.f3.pdf]
